# Hepatic schistosomiasis, upper gastrointestinal bleeding, and health related quality of life measurements from the Albert Nile Basin

**DOI:** 10.1186/s41687-021-00389-9

**Published:** 2021-10-30

**Authors:** Christopher K. Opio, Francis Kazibwe, Lalitha Rejani, Narcis B. Kabatereine, Ponsiano Ocama

**Affiliations:** 1 Aga Khan University Hospital, 3rd Parkland Avenue, PO Box 30270-00100, Nairobi, Kenya; 2 Public Health Department, Bishop Stuart University, PO Box 9, Mbarara, Uganda; 3 Makerere University College of Health Sciences, PO Box 7072, Kampala, Uganda; 4 Vector Control Division, Ministry of Health, Kampala, Uganda

**Keywords:** Health related quality of life measurements, Disability weights, Upper gastrointestinal bleeding, Schistosomiasis, EQ-5D/EQ-VAS

## Abstract

**Background:**

Health related quality of life measurements are vital elements of public health surveillance that uncover unmet health needs and predict the success of health interventions. We described health related quality of life measurements using the EuroQoL 5-dimension (EQ-VAS/EQ-5D) instrument and associated factors among patients with upper gastrointestinal bleeding (UGIB) and hepatic schistosomiasis at a rural health facility in the Albert Nile Basin, Uganda.

**Methods and materials:**

This was a cross-sectional study at Pakwach Health Centre IV. Participants included adult inpatients and outpatients with a history of UGIB and ultrasound evidence of hepatic schistosomiasis. We evaluated and recorded each participant’s medical history, physical examination, laboratory tests results, ultrasound results, and endoscopy findings. We also recorded health related quality of life measurements using the EuroQoL 5-dimension instrument and derived disability weights from EQ-VAS and EQ-5D measurements. These were our dependent variables. Descriptive and inferential statistics were generated summarizing our findings.

**Results:**

We found 103 participants had a history of upper gastrointestinal bleeding and hepatosplenic schistosomiasis. Sixty percent were between the ages of 30–49 years, 59% were females, 74% were farmers, 92% had splenomegaly, 88% had varices at endoscopy, 22% were medical emergencies with acute variceal upper gastrointestinal bleeding, and 62% had anemia. Measures of the different dimensions of health from 101 participants with patient reported outcomes revealed 77 (76%) participants experienced problems in self-care, 89 (88%) participants reported anxiety or depression, and 89 (88%) participants experienced pain or discomfort. The median EQ-VAS derived disability weights and median EQ-5D index-derived disability weights were 0.3 and 0.34, respectively. Acute upper gastrointestinal bleeding, praziquantel drug treatment, and age by decade predicted higher EQ-VAS derived disability weights (*p* value < 0.05). Under weight (Body mass index ≤ 18.5), acute upper gastrointestinal bleeding, ascites, age by decade, female gender, and praziquantel drug treatment predicted higher EQ-5D index- derived disability weights (*p* value < 0.05).

**Conclusion:**

Adult patients with upper gastrointestinal bleeding and hepatic schistosomiasis from this primary health facility experience poor health and considerable health loss. Several factors predicted increased health loss. These factors probably represent key areas of health intervention towards mitigating increased health loss in this population.

**Supplementary Information:**

The online version contains supplementary material available at 10.1186/s41687-021-00389-9.

## Background

Dr. Warren in 1978 termed hepatic schistosomiasis, the “great neglected disease of the liver”. Hepatic schistosomiasis is an insidious disease that follows embolization of schistosoma eggs to the liver and portal system, leading to chronic inflammation, tissue injury, and progressive tissue fibrosis [1, 2]. Extensive periportal fibrosis, portal hypertension, varices, upper gastrointestinal bleeding, splenomegaly, and ascites are the hallmarks of advanced disease. Variceal upper gastrointestinal bleeding is the principal cause of morbidity, hospital admissions, and death among individuals with advanced hepatic schistosomiasis [3–8].

Chronic infection with *S. mansoni* is the only cause of hepatic schistosomiasis in Sub-Saharan Africa. In Uganda, the Albert Nile basin is a hot spot for *S. mansoni* infection [9, 10]. Studies show that between 30% to 50% of persons infected with *S. mansoni* from the Albert Nile basin had hepatic schistosomiasis [11–16]. Studies also show that 20% to 50% of adult patients diagnosed with hepatic schistosomiasis from an endemic area will also have portal hypertension, varices, variceal upper gastrointestinal bleeding at the time of first diagnosis [6, 8].

Currently Praziquantel mass drug administration is the main control intervention for schistosomiasis worldwide. However, current evidence shows that Praziquantel mass drug administration has failed. Many Countries in Sub-Saharan Africa, including Uganda, did  not meet the World Health Organization’s goal for control and elimination of schistosomiasis by the year 2020 [17–20].

In Uganda, limited health financing has restricted schistosomiasis control measures to surveillance and Praziquantel mass drug administration [21, 22]. As a result, other aspects of control and care of patients with hepatic schistosomiasis seem neglected [23, 24]. Nowadays, there is no new data about the morbidity, mortality, and quality of life of patients with upper gastrointestinal bleeding and hepatic schistosomiasis from the Albert Nile basin. It is still not clear what health problems patients with upper gastrointestinal bleeding and hepatic schistosomiasis from the Albert Nile Basin face. We also do not know what perceptions they have about their health status as they seek healthcare.

Measures of the health status of individuals and populations are essential for any evidence-based health policy. Health related quality of life measurements are also vital elements of public health surveillance. Health related quality of life measurements can uncover unmet health needs and predict the success of health interventions [25, 26]. However, patients may experience unmet needs that based on their clinical profile or on various clinical factors.

Health related quality of life measurements are important for successful control of hepatic schistosomiasis and upper gastrointestinal bleeding in the Albert Nile Basin. While clinical factors associated health related quality of life measurements are important for identifying unmet needs in clinical care.

We described health related quality of life measurements using the EQ-5D tool and associated clinical factors among patients with upper gastrointestinal bleeding and hepatic schistosomiasis at a rural health facility in the Albert Nile Basin.

## Materials and methods

This was a cross-sectional study that involved individuals attending the outpatient department and inpatients admitted acutely with severe upper gastrointestinal bleeding at the same health facility. The study was conducted over 6 weeks between the months of July and August 2014 at Pakwach Health Centre IV, a government run primary health care facility located on the banks of the Albert Nile in northwestern Uganda. Pakwach Health Centre provides outpatient and inpatient health care services to a population who are mainly fisher persons and/or farmers. Epidemiological data suggests close to 50% of the populations served by this facility are infected with *S. mansoni* despite over a decade of Praziquantel chemotherapy. Medical records from the facility indicated about 120 patients every year are admitted for acute upper gastrointestinal bleeding as a complication of chronic schistosomiasis.

Eligibility for study participation included written informed consent and ascent, all patients ≥ 12 years of age with a medical history of upper gastrointestinal bleeding (past or current). We excluded pregnant women, HIV positive individuals, and any participant unable to have endoscopy for upper gastrointestinal bleeding. Pregnant women were excluded because of the restrictions of undertaking endoscopy. HIV patients were excluded from this study because ethical considerations (confidentiality and partner disclosure) and the fact that another study was already recruiting patients with HIV and schistosomiasis [7, 27].

Upper gastrointestinal bleeding was defined by any lifetime history of hematemesis, melena, or hematochezia. Participants at the outpatient’s department were systematically sampled and enrolled, while inpatients were consecutively recruited over 6 weeks. A detailed medical history that included socio-demographic data, exposure to schistosoma species or alcohol, treatment of schistosomiasis and time from the last treatment, history of upper gastrointestinal bleeding, and other relevant past medical history. Participants were examined for stigmata of chronic liver disease, and vital signs [7, 27].

Measures of health-related quality of life were obtained using the three-level European Quality of Life 5-Dimensions (EQ-5D-Y) questionnaire and the visual analogue scale (EQ-VAS) from the EuroQol Group (EQ-5D™) [28, 29]. The EQ-5D-Y is a multi-attribute utility instrument. The EQ-5D represents measures of personal well-being and this indicates opinions referenced to the general population. It has 5 dimensions that include mobility, self-care, usual activities, pain/discomfort, and anxiety/depression. Each dimension is rated as a Likert scale from having no problems (level 1), some problems (level 2), to extreme problems (level 3). The EQ-5D data was converted into an index of health (EQ-5D index) using crosswalk values from Zimbabwe in Stata [30]. These were considered the closest values to our study population. Zimbabwe is found in south sub-Saharan Africa and schistosomiasis is endemic in Zimbabwe [31]. The economic and health indices are comparable. The EQ-5D index represents a measure of health from 0 for death to 1 for perfect health. The EQ-VAS is a visual analogue scale (VAS). The scale varies from 0, the worst imaginable health state, to 100, the best imaginable health state. The EQ-VAS asks patients to indicate their overall health on a vertical visual analogue scale (0 to 100). This indicates opinions referenced to an individual. The EQ-VAS and EQ-5D index were then transformed to disability weights by the formulas [EQVAS-derived disability weights = 1 − (VAS/100)] and [EQ-5D index-derived disability weights = 1 − EQ-5D index] respectively. A disability weight is a weight factor that reflects the severity of the disease on a scale from 0 (perfect health) to 1 (equivalent to death). Disability weights characterize the amount of health loss associated with specific health outcomes and are used to calculate years living with disability [28, 29, 32–35].

Blood was analyzed generating 3-part hematology indices (using a compact Sysmex KX-21 hematology analyzer), hepatitis B and C viral blood serology results (obtained from commercially available rapid diagnostic test kits), and malaria antigen test results (from rapid diagnostic test kits). Stool microscopy was performed for Ova and urine for schistosomiasis using the urine circulating cathodic antigen (CCA) test by Rapid Diagnostics. Trans-abdominal ultrasonography was performed by a trained sonographer according to the modified World Health Organization Niamey protocol using the SONOSTAR model SS8, a portable ultrasound with a 3.5 MHz convex probe. Upper digestive endoscopy was performed using a Pentax EPKi digital video processor and a Pentax 9.8 mm video gastroscope after a local anesthetic (Xylocaine spray) by a gastroenterologist.

Data was transcribed from questionnaires and entered to Microsoft Access database. This was edited to ensure quality and exported to Stata version 16 (STATA Corp, Lakeway, College Station, Texas, USA).

Descriptive and inferential statistics were generated. These described the study population and measures of health-related quality of life (EQ-VAS, EQ-5D dimension, EQ-5D index, EQ-VAS-derived disability weights, and EQ-5D index-derived disability weights). Clinical factors associated with EQ-VAS-derived disability weights, and EQ-5D index-derived disability weights were also explored. Categorical data were summarized as proportions with logit-transformed 95% confidence intervals. Continuous data were summarized as medians, interquartile range (IQR), and 95% confidence intervals. Medians and their 95% confidence intervals were generated using binomial method that makes no assumptions about the underlying distribution of the variable.

The strength and direction of relationship between EQ-5D dimensions, EQ-VAS, EQ-5D index, EQ-VAS-derived disability weights, and EQ-5D index-derived disability weights were explored by Spearman’s correlation coefficient. This is summarized in a table format.

Multivariable analysis involved disability weights (EQ-VAS-derived disability weights and EQ-5D index-derived disability weights) as the dependent variables. We designated clinical variables highlighted in Table 4 as independent variables. These included the following variables age range, gender, occupations (farmer and fishermen), body mass index, ascites, splenomegaly, edema, jaundice, esophageal varices, acute upper gastrointestinal bleeding, urine CCA for detection of schistosomiasis, hepatitis B surface antigen positivity, and previous treatment with praziquantel. Lasso in Stata 16 was used to select covariates and models. This process included cross validation, adaptive lasso, plug in’s for heteroskedasticity and homoskedasticity. We selected best regression models based on “Goodness of fit” after Lasso. Considering error variance, we performed heteroskedasticity linear regression for EQ-VAS-derived disability weights as the dependent variable. Homoskedasticity linear regression was performed for EQ-5D index-disability weights as the dependent. The models were subject to population averaged marginal effects analysis for all covariates in Stata. A significance level (*p* value < 0.05) was considered for all analysis, and confidence intervals or standard errors supported inference. We summarized these results as text, in tables, and as figures.

### Ethics statement

This was a cross-sectional study that involved human participants. School of Medicine, Makerere University, Institutional review board, Kampala, Uganda (#REC REF2011-244), and the Uganda National Council for Science and Technology, Kampala, Uganda (UNCST approval #, HS 1620) approved this study. The study was conducted according to the principles expressed in the Declaration of Helsinki. Written informed consent was obtained from all participants.

## Results

All participants had a past or current medical history of upper gastrointestinal bleeding. One fifth (23 participants out of 107 enrolled) presented as medical emergencies and were admitted with acute severe upper gastrointestinal bleeding over the study period. We screened 324 at the outpatient’s department over the same period and were able to enroll 84 patients with a past medical history of upper gastrointestinal bleeding.

Four out of the 107 participants did not have any evidence of hepatic schistosomiasis after ultrasonography. Of 103 participants who had hepatic schistosomiasis, 2 participants had missing EQ-5DY records. The youngest participant was 25 years and the oldest was 71 years. Sixty percent of our study population were aged 30–49 years. The female-male ratio was 7 to 5. Most participants were long-term residents of Pakwach. All participants had frequent contact with the waters of the Nile (5–7 times a week) and nearly all were either fisherpersons or farmers by occupation. Among our participants, 55% had a prior diagnosis of intestinal schistosomiasis, 88% had ever received Praziquantel in the past, 8% had active infection at the time of enrolment, 92% had splenomegaly on physical exam and 96% had moderate to severe periportal fibrosis and/or cirrhosis at ultrasonography. All reported experiencing upper gastrointestinal bleeding with 96% reporting at least one lifetime episode of hematemesis, and 97% reporting past admission for upper gastrointestinal bleeding. No participant has ever had an endoscopy for upper gastrointestinal bleeding nor was on propranolol for prevention of recurrent variceal bleeding. Endoscopy was performed on all 107 participants during the study. Following endoscopy, we found 86 had varices, 8 had both varices and peptic ulcers, and 21 had peptic ulcers alone. Among the 103 participants who had hepatic schistosomiasis, 85 (83%) had varices and 18 (18%) had peptic ulcer disease. The clinical, laboratory, ultrasound, and endoscopic characteristics of the 103 participants, proportions and their 95% confidence intervals are summarized in Table 1.

**Table 1:** Clinical, laboratory, ultrasound, and endoscopic characteristics of adult patients with hepatosplenic schistosomiasis and upper gastrointestinal bleeding

N = 103^a^	Percentage	Logit confidence limits	
		Lower limit (5%)	Upper limit (95%)
*Age ranges*			
Age = 18–29 years	4	1	10
Age = 30–39 years	24	17	34
Age = 40–49 years	36	27	46
Age = 50–59 years	18	12	27
Age = 60–69 years	15	9	23
Age ≥ 70 years	3	1	9
*Gender*			
Male	41	32	51
Female	59	49	68
*Stayed in Pakwach for over 10 years*			
No	12	7	20
Yes	88	80	93
*Contact with the Nile 5 to 7 times a week*			
No	1	0	7
Yes	99	93	100
*Farmer*			
No	26	19	36
Yes	74	64	81
*Fisherman*			
No	84	76	90
Yes	16	10	24
*Hematemesis*			
No	4	1	10
Yes	96	90	99
*Melena stool*			
No	55	46	65
Yes	45	35	54
*Hematochezia*			
No	49	39	58
Yes	51	42	61
*Schistosomiasis by positive stool test in the past*			
No	45	35	54
Yes	55	46	65
*Praziquantel*			
No	12	7	20
Yes	88	80	93
*Ever admitted for upper gastrointestinal bleeding*			
No	3	1	9
Yes	97	91	99
*Ever received a blood transfusion*			
No	30	22	40
Yes	70	60	78
*Splenomegaly*	(physical examination)		
No	8	4	16
Yes	92	84	96
*Ascites*	(physical examination)		
No	83	74	89
Yes	17	11	26
*Jaundice*	(physical examination)		
No	89	82	94
Yes	11	6	18
*Edema*	(physical examination)		
No	88	80	93
Yes	12	7	20
*Body mass index*			
≤ 18.5	17	10	25
18.5–24.9	79	70	86
25–29.9	4	2	11
*Acute Severe variceal upper gastrointestinal bleeding*			
No	78	69	85
Yes	22	15	31
*Fibrosis patterns*			
C	5	2	11
D	33	25	43
E	20	14	29
F	4	1	10
X	38	29	48
*Spleen length* ≥ *15 cm*			
No	40	31	50
Yes	60	50	69
*Portal vein* ≥ *13 mm*			
No	23	16	33
Yes	77	67	84
*Ascites at ultrasound*			
No	85	77	91
Yes	15	9	23
*Anemia grade(gm/dl)*			
< 6.5	19	13	28
6.5–7.9	11	7	20
8–9.4	15	9	23
9.5–10.9	17	10	25
≥ 11	38	29	48
*Hepatitis B surface antigen positive*			
No	93	86	97
Yes	7	3	14
*Hepatitis C antibody positive*			
No	74	64	81
Yes	26	19	36
*Urine CCA*			
No	92	85	96
Yes	8	4	15
*Varices*			
No	17	11	26
Yes	83	74	89

^a^Two participants had incomplete EQ5D records from the 103 participants

### Health related quality of life measurements

**Fig.1:**
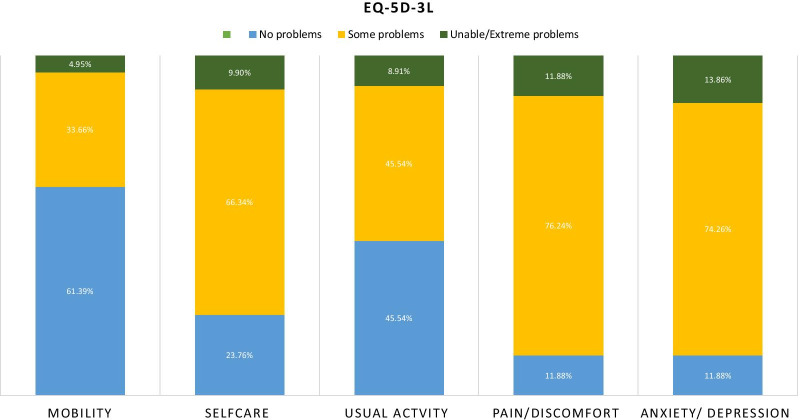
Response distribution of EQ5D-3 level domains for participants with upper gastrointestinal bleeding and schistosomiasis (n = 101)

Measures of the 5 dimensions of health revealed 77 (76%) participants had some or extreme problems in self-care, 89 (88%) participants reported some or extreme anxiety or depression, and 90 (89%) participants reported experiencing some or extreme pain or discomfort. Only 37 (37%) participants reported some or extreme problems in their mobility. However, 55 (55%) participants were able to undertake their usual activities without any problems (Fig. 1).

Table 2 summarizes calculated medians with confidence intervals for the EQ visual analogue scale measurements, EQ-5D utility index, EQ visual analogue scale derived disability weights, and EQ-5D utility index derived disability weights by different response variables. Covariates of interest had a median EQ visual analogue scale derived disability weight > 0.40 or a median EQ-5D utility index derived disability weight > 0.35.

**Table 2:** Medians (95% confidence intervals) of EQ-VAS, EQ-5D index, and derived disability weights by different clinical factors

	n	EQ-VAS		EQ-5D index		EQ-VAS derived disability weights		EQ-5D index derived disability weights	
		Median	95% CI	Median	95% CI	Median	95% CI	Median	95% CI
All participants	101	70	60, 70	0.59	0.59, 0.62	0.30	0.30, 0.40	0.34	0.31, 0.35
*Age, years*									
18–29	4	75	70, 90	0.67	0.59, 0.73	0.25	0.10, 0.30	0.26	0.21, 0.35
30–39	25	70	60, 80	0.62	0.52, 0.62	0.30	0.20, 0.40	0.31	0.31, 0.38
40–49	36	65	50, 70	0.62	0.59, 0.62	0.35	0.30, 0.50	0.31	0.31, 0.35
50–59	18	50	40, 80	0.59	0.09, 0.73	0.50	0.20, 0.60	0.38	0.31, 0.53
60–69	15	60	50, 70	0.52	0.19, 0.62	0.40	0.30, 0.50	0.40	0.31, 0.50
≥ 70	3	30	20, 60	− 0.13	− 0.18, − 0.13	0.70	0.40, 0.80	0.78	0.77, 0.78
Men	40	70	50, 80	0.62	0.59, 0.66	0.30	0.20, 0.50	0.31	0.30, 0.35
Women	61	60	50, 70	0.55	0.52, 0.62	0.40	0.30, 0.50	0.36	0.31, 0.40
Fisherman	14	70	50, 80	0.68	0.58, 0.79	0.30	0.20, 0.50	0.33	0.21, 0.42
Farmer	76	70	60, 70	0.65	0.62, 0.70	0.30	0.30, 0.40	0.35	0.31, 0.38
*Jaundice*									
No	90	70	60, 70	0.62	0.59, 0.62	0.30	0.30, 0.40	0.31	0.31, 0.35
Yes	11	60	40, 70	0.52	− 0.16, 0.59	0.40	0.30, 0.60	0.40	0.34, 0.68
*Edema*									
No	90	70	60, 70	0.62	0.59, 0.62	0.30	0.30, 0.40	0.31	0.31, 0.35
Yes	11	40	30, 50	0.15	− 0.18, 0.62	0.60	0.50, 0.70	0.48	0.31, 0.77
*Splenomegaly*									
No	8	65	20, 90	0.57	− 0.35, 0.80	0.35	0.10, 0.80	0.36	0.17, 0.99
Yes	89	70	60, 70	0.62	0.59, 0.62	0.30	0.30, 0.40	0.32	0.31, 0.35
Tense ascites (exam difficult)	4	30	10, 40	0.12	− 0.59, 0.15	0.70	0.60, 0.90	0.51	0.48, 1.15
*Praziquantel*									
No	11	80	70, 90	0.66	0.52, 0.80	0.2	0.10, 0.30	0.30	0.17, 0.34
Yes	90	60	50, 70	0.59	0.52, 0.62	0.4	0.30, 0.50	0.35	0.31, 0.40
*Body mass index ≤ 18.5*									
No	84	70	60, 70	0.62	0.59, 0.62	0.3	0.30, 0.40	0.31	0.31, 0.35
Yes	17	50	40, 70	0.52	1.66, 0.62	0.5	0.30, 0.60	0.41	0.31, 0.78
*Liver fibrosis pattern score*									
C	5	60	50, 90	0.59	− 0.13, 0.70	0.4	0.10, 0.50	0.35	0.21–0.78
D	34	70	60, 80	0.62	0.55, 0.66	0.3	0.20, 0.40	0.31	0.30, 0.35
E	21	70	50, 80	0.62	0.52, 0.62	0.3	0.20, 0.50	0.32	0.31, 0.40
F	3	50	50, 80	0.59	0.52, 0.62	0.5	0.20, 0.50	0.36	0.31, 0.40
X	38	60	50, 70	0.59	0.24, 0.62	0.35	0.31, 0.42	0.35	0.31, 0.42
*Ascites by ultrasound*									
No	87	70	60, 70	0.62	0.59, 0.62	0.3	0.30,0.40	0.31	0.31, 0.35
Yes	14	40	30, 50	0.15	− 1.26, 0.52	0.6	0.50, 0.70	0.48	0.40, 0.77
*Varices*									
No	18	70	50, 80	0.6	0.52, 0.67	0.3	0.20, 0.50	0.33	0.30, 0.40
Yes	83	70	50, 70	0.59	0.52, 0.63	0.3	0.30, 0.50	0.35	0.31, 0.36
*Acute upper gastrointestinal bleeding*									
No	79	70	70, 80	0.62	0.59, 0.62	0.3	0.20, 0.30	0.31	0.31, 0.35
Yes	22	40	30, 50	0.2	− 0.13, 0.59	0.6	0.50, 0.70	0.45	0.36, 0.68
*Urine CCA*									
No	93	70	60, 70	0.59	0.52, 0.62	0.3	0.30,0.40	0.35	0.31, 0.36
Yes	8	65	0, 80	0.6	− 0.13, 0.62	0.35	0.20, 1.0	0.33	0.31, 0.78
*Anemia grade (gm/dl)*									
< 6.5	20	50	40, 70	0.6	0.29, 0.64	0.5	0.30, 0.60	0.40	0.36, 0.71
6.5–7.9	12	50	31, 69	0.56	0.47, 0.55	0.5	0.31, 0.69	0.44	0.35, 0.53
8.0–9.4	15	60	50, 80	0.65	0.60, 0.70	0.4	0.20, 0.50	0.35	0.31, 0.40
9.5–10.9	16	65	55, 80	0.72	0.62, 0.79	0.35	0.20, 0.45	0.28	0.21, 0.38
≥ 11.0	38	75	70, 80	0.7	0.69, 0.70	0.25	0.20, 0.30	0.31	0.30, 0.31
*Hepatitis B surface antigen positive*									
No	94	60	50, 70	0.65	0.62, 0.70	0.4	0.30, 0.50	0.35	0.31, 0.38
Yes	7	80	70, 87	0.7	0.65, 0.79	0.2	0.13, 0.30	0.31	0.21, 0.35

UGIB-upper gastrointestinal bleeding, 95CI – 95% confidence intervals

Table 3 summarizes spearman’s rank correlation coefficients and significance levels (*p* value < 0.05) for all the 5 dimensions of the EQ-5D questionnaire, EQ visual analogue scale, EQ-5D utility index, EQ visual analogue scale derived disability weights, and EQ-5D utility index derived disability weights. EQ-5D index was positively and very strongly correlated with EQ-VAS (Spearman’s rank correlation, n = 101, Rho = 0.73, *p* value = 0.0001). A guide enabling inference of the correlation coefficients’ is provided within the table (interpretation of effect size) [36]. All parameters showed moderate to very large correlations with each other (0.30 to 0.9).

**Table 3:** Spearman’s rank correlation coefficients of EQ-5 domains, EQ-VAS, EQ-5D index and derived disability weights

	Mobility	Self-care	Usual activities,	Pain and discomfort	Anxiety and depression	EQ-VAS	EQ-5D index	EQ-VAS derived disability weights	EQ-5D Index derived disability weights
Mobility	1								
Self-care	0.3916*	1							
Usual activities,	0.5849*	0.4725*	1						
Pain/discomfort	0.4721*	0.3898*	0.4550*	1					
Anxiety/depression	0.3364*	0.3429*	0.2253	0.3151*	1				
EQ-VAS	− 0.6308*	− 0.4928*	− 0.6051*	− 0.4300*	− 0.4162*	1			
EQ-5D index	− 0.7553*	− 0.7494*	− 0.7160*	− 0.6270*	− 0.5922*	0.7288*	1		
EQ-VAS disability weight	0.6308*	0.4928*	0.6051*	0.4300*	0.4162*	− 1	− 0.7288*	1	
EQ-5D disability weight	0.7553*	0.7494*	0.7160*	0.6270*	0.5922*	− 0.7288*	− 1	0.7288*	1

# Correlation coefficient	Interpretation of effect size
.90 to 1.00	Nearly, practically, or almost: perfect, distinct, infinite
.70 to .90	Very large, very high, huge
.50 to .70	Large, high, major
.30 to .50	Moderate, medium
.10 to .30	Small, low, minor
.00 to .10	Trivial, very small, insubstantial, tiny, practically zero

^*^Significance level *p* < 0.05

### Averaged marginal effects for all covariates for EQ visual analogue scale derived disability weights, and EQ-5D utility index derived disability weights

**Fig. 2:**
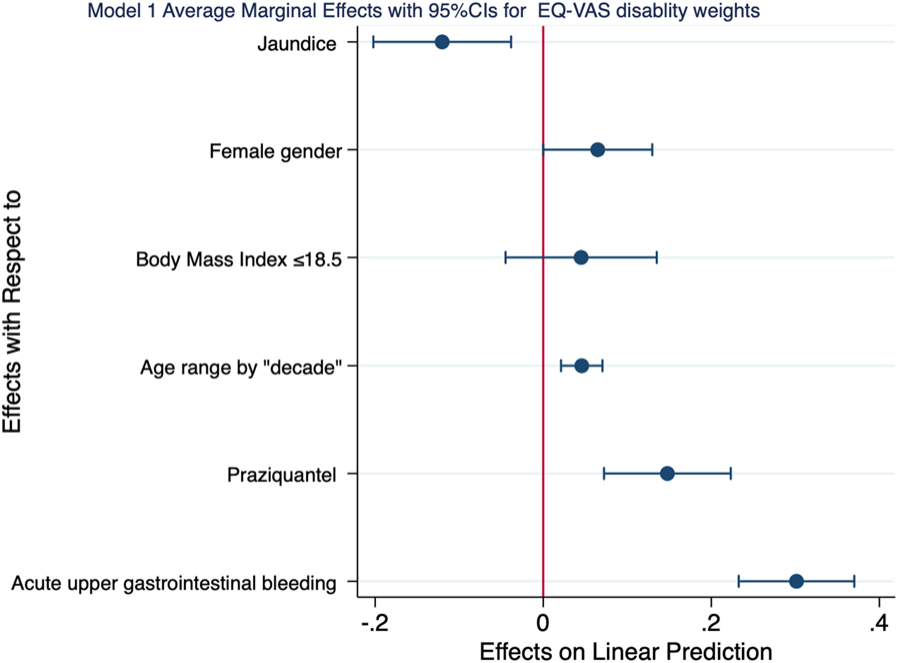
Average marginal effects for different covariate on EQ-VAS derived disability weights

Our results demonstrated that the probability of EQ-VAS derived disability weights increased by 30 percentage points, 15 percentage points, 5 percentage points, and 7 percentage points when one experienced acute upper gastrointestinal bleeding, received praziquantel drug treatment, for every age by decade, and if one were of female gender respectively. While the probability of EQ-VAS derived disability weights decreased by 12 percentage points when one had Jaundice. All these changes were statistically significant (Table 4 and Fig. 2).

**Table 4:** Summarizes the average marginal effects for EQ-VAS derived disability weights and EQ-5D derived disability weights by different covariates after linear regression and marginal analysis

	Delta-method		
	dy/dx	95% Confidence Intervals	*p* value
*Model 1—EQ-VAS derived disability weights*			
Acute upper gastrointestinal bleeding	0.30	0.23, 0.37	0.001
Praziquantel	0.15	0.07, 0.22	0.001
Age range by decade	0.05	0.02, 0.07	0.001
Body Mass Index ≤ 18.5	0.05	− 0.05, 0.14	0.326
Female gender	0.07	0.00002, 0.13	0.050
Jaundice	− 0.12	− 0.20, − 0.04	0.004
*Model 2—EQ-5D derived disability weights*			
Body mass index ≤ 18.5	0.19	0.05, 0.33	0.009
Acute upper gastrointestinal bleeding	0.10	0.02, 0.18	0.018
Ascites	0.19	0.05, 0.32	0.010
Age range by decade	0.03	0.003, 0.06	0.031
Female gender	0.10	0.03, 0.18	0.006
Praziquantel	0.12	0.04, 0.20	0.003
Farmers	0.07	− 0.005, 0.14	0.069
World Health Organization Anemia grade	− 0.007	− 0.04, 0.03	0.690

Marginal analysis enables examination of the impact of variable x (covariates) on outcome y (disability weights) for representative cases

**Fig. 3:**
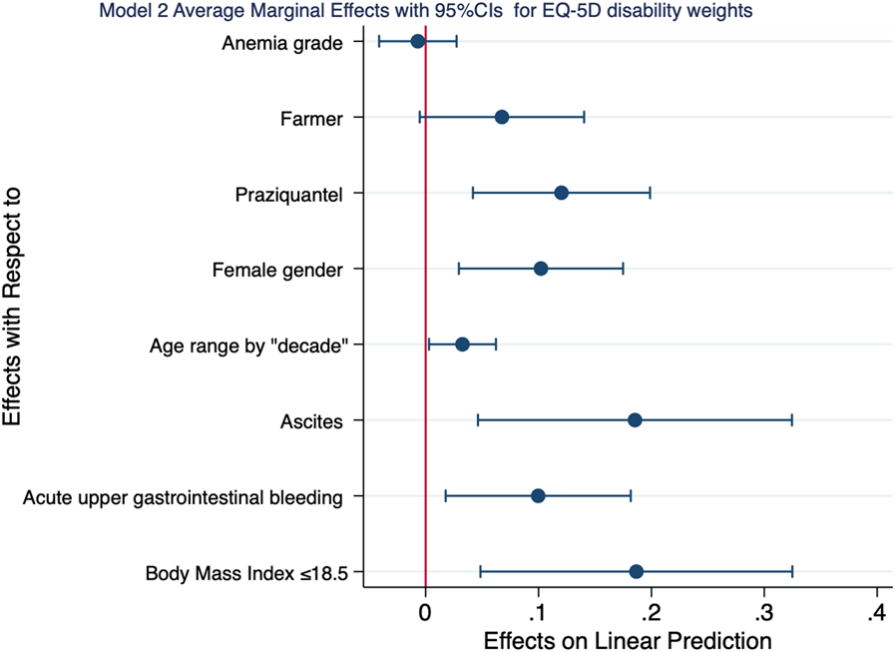
Average marginal effects for different covariate on EQ-5D index derived disability weights

We also demonstrated that probability of EQ-5D index derived disability weights increased by 19 percentage points, 10 percentage points, 19 percentage points, 03 percentage points, 10 percentage points, and 12 percentage points when one was under weight (BMI ≤ 18.5), experienced acute upper gastrointestinal bleeding, one had ascites, for every age by decade, if one was of female gender, and had praziquantel drug treatment respectively. All these changes were statistically significant. All these changes were statistically significant (Table 4 and Fig. 3).

## Discussion

Our study reports about EQ-5D derived health-related quality of life measurements from patients with hepatic schistosomiasis and upper gastrointestinal bleeding at a rural health facility in the Albert Nile basin. As far as we know, few studies have published EQ-5D outcome measurements about patients with hepatic schistosomiasis. One study from Brazil and involved patients with *S. mansoni* infection [34]. The other study from China involved patients with *S. japonica* infection [32]. Our study is unique because it is from Sub-Saharan Africa, where 90% of those who have schistosomiasis worldwide reside. It is also distinct because we focused only on patients with any life history of upper gastrointestinal bleeding at a rural primary health care facility in rural Africa. Primary health care research is essential for improved health policy [37].

The characteristics of our participants are not unique. Daily contact with the waters of the Nile, periportal fibrosis, splenomegaly, and varices were frequent in our study participants. This is in keeping with published literature [13, 16, 38–40]. The differential diagnosis of such a clinical profile is liver cirrhosis. However, liver cirrhosis was less likely because of the low prevalence of alcohol misuse and viral hepatitis B or C infections in our study [7]. We also found a low prevalence of stigmata of chronic liver disease including ascites, hepatic encephalopathy, and jaundice. These stigmata are more frequent in liver cirrhosis than hepatosplenic schistosomiasis [40, 41]. Peptic ulcer disease was infrequent and was less likely to cause upper gastrointestinal bleeding among our participants. Regarding care, none of our participants had endoscopy, and no participant with varices was on propranolol to prevent recurrent variceal bleeding before enrollment into the study. Current care guides recommend endoscopy for all with upper gastrointestinal bleeding and/or propranolol for those with upper gastrointestinal bleeding and varices [4, 42, 43].

Evaluation of EQ-5D primary data showed that our participants reported more problems in all the five measured health domains (mobility, self-care, usual activities, pain/discomfort, and anxiety/depression) than the study by Nascent et al. In addition, the mean and median EQ-VAS scores in our study were also lower than the study by Nascent et al. [34]. These differences were greatest for the domains of welfare, and anxiety/depression This could be explained by dissimilarities in disease severity and access to better health care services.

Jia et al. reported similar findings for dimensions of pain/discomfort, anxiety/ depression, and mobility. However, differences are apparent for dimensions of usual activities and self-care. The high prevalence of ascites from that Jia et al. could explain this variance [32].

Our EQ-5D measurements (index and VAS) are comparable with a systematic review about health state estimates about diseases [44]. From our study, the EQ-5D index and EQ-5D VAS were strongly positively correlated. This indicates that using Zimbabwe cross talk values did not adversely impact our EQ-5D index estimates. The average disability weights EQ-5D VAS derived disability weights, and EQ-5D index derived disability weights from our study population were 0.3 and 0.34 respectively. This suggests that our participants experienced poor health 4 months out of 12 months for each survived life year. The “Global Burden of Disease Study 2017” has reported disability weights for hematemesis, severe anemia, and ascites from schistosomiasis as 0.325, 0.149, and 0.144 respectively [35]. Nearly all our patients reported a history of hematemesis. The average EQ-5D VAS derived disability weights, and EQ-5D index derived disability weights from our study population were 0.3 and 0.34 respectively. These are like what are reported by the “Global Burden of Disease Study 2017”. On the other hand, disability weights for severe anemia, and ascites from the “Global Burden of Disease Study 2017” are 3 to 4 times less than what we found from our study. The “Global Burden of Disease Study 2017” did not provide disability weights for acute variceal upper gastrointestinal bleeding [35]. The implications of some of these findings have been discussed by Jia et al. [32]. We echo similar views.

Factors associated with EQ-5D VAS derived disability weights included acute upper gastrointestinal bleeding, Praziquantel drug treatment, age range by decade, and female gender. While factors associated with EQ-5D index derived disability weights included underweight (BMI ≤ 18.5), acute upper gastrointestinal bleeding, ascites, age range by decade, female gender, and Praziquantel drug treatment. Jia et al., reported that EQ-5D VAS derived disability weights were associated with age, impaired work capacity, depression, anxiety, ascites, and active hepatitis B infection. We found similar findings for, depression/anxiety, ascites, and active hepatitis B infection (Additional file 1). The negative coefficient in the model could be explained by the inadvertent exclusion of patients with hepatitis B virus decompensated liver disease i.e., jaundice, Additional file 1.

To the best of our knowledge, no study has directly linked the occurrence of acute upper gastrointestinal bleeding and schistosomiasis to higher disability. What is known is that acute upper gastrointestinal bleeding is the main reason for hospitalization and death among patients with schistosomiasis due to *S. mansoni* [5, 6, 45, 46]. It therefore stands to reason that preventing acute upper gastrointestinal bleeding could decrease health loss in this population [8]. In the same way, addressing ascites and praziquantel adverse drug effects in this population could also decrease health loss. These assertions are supported by published research which shows that many acute illnesses requiring hospitalization are usually associated with increased disability. Preventing hospitalization decreases disability or health loss [47]. Acute upper gastrointestinal bleeding can be prevented through secondary prophylaxis with drugs, bands, and shunts. Ascites can be treated/prevented with drugs and paracentesis.

Praziquantel treatment has been associated with adverse drug effects including occurrence recurrent and acute upper gastrointestinal bleeding in this population [48, 49]. Mitigating these adverse effects could improve health related quality of life measurements in this study population.

One retrospective case series reported a significantly higher proportion of females presented with upper gastrointestinal bleeding due to varices than males (39.4% vs. 23.6%, *p* < 0.001) [50]. Research from this study population showed that female gender was a predictor of recurrent upper gastrointestinal bleeding [7]. We propose that there is a link between female gender, recurrent upper gastrointestinal bleeding, and disability. Nascent et al., reported that median EQ-5D index values from their study was significantly lower among females and patients with comorbidities [34]. Underweight (body mass index ≤ 18.5) has been associated with poor self-rated health [51].

Our study has limitations. Our sample size is small, and this was a single Centre cross sectional study at a rural primary health care facility. This makes our results less conclusive for several reasons. Small studies are likely to overestimate the magnitude of association. Furthermore, we could have inadvertently excluded individuals in this community that do not have access to this facility for any reason including death due to acute upper gastrointestinal bleeding. This is evident from the direction of association between jaundice and disability.

We also acknowledge that our use of the EQ-5D (a generic quality of life measurement tool) and the lack of population-specific crosstalk data from the Albert Nile basin could have made our EQ-5D index-disability measurements less precise. The EQ-5D index could have introduced an exogenous source of variance. Nevertheless, we found many similarities between our study and what has been published by others about health related quality of life measurements from patients with hepatic schistosomiasis [32, 34, 35, 52]. This suggests that our results might be more robust than we demonstrate.

## Conclusions

Adult patients with upper gastrointestinal bleeding and hepatic schistosomiasis from this rural primary health facility in sub–Saharan Africa experienced poor health and substantial health loss. These findings are comparable with studies from china and brazil about hepatic schistosomiasis. We also identified several factors associated with individual health loss among participants with upper gastrointestinal bleeding and schistosomiasis. These factors probably represent unmet health needs and may point out potential areas for health intervention. Even so, more research is needed to confirm these findings.

## Supplementary Information

**Additional file 1.** Ancillary descriptive and inferential results.

**Additional file 2.** De-identified data set.

## Data Availability

Data is available and is provided as Additional file 2.
