# Supplementary material for: Hepatic schistosomiasis, upper gastrointestinal bleeding, and health related quality of life measurements from the Albert Nile Basin
Source: J Patient Rep Outcomes. 2021 Oct 30;5:112. doi: 10.1186/s41687-021-00389-9 (PMC8557235; doi:10.1186/s41687-021-00389-9)
Supplement: Supplementary file 1 — Additional file 1. Ancillary descriptive and inferential results. [file 41687_2021_389_MOESM1_ESM.docx]

Supplementary Tables 1,2,3,4, and 5.

Supplementary Table 1 Frequency tabulation of pain domains by mobility

|  | M123 |  |  |  |
| --- | --- | --- | --- | --- |
| PAIN123 | 1 | 2 | 3 | Total |
|  |  |  |  |  |
| 1 | 11 | 1 | 0 | 12 |
| 2 | 51 | 24 | 2 | 77 |
| 3 | 0 | 9 | 3 | 12 |
|  |  |  |  |  |
| Total | 62 | 34 | 5 | 101 |

Supplementary Table 2 Medians (centiles) and confidence intervals for the different health related quality of life measurements tabulated by the absence or presence of hepatitis B infection or Jaundice.

*EQvas- EQVAS, _index- EQ5D index, DW1- EQVAS disability weight, DW2- EQ5D disability weight.*

*HBV – hepatitis B surface antigen positive, JAUNDICE –Jaundice*

by HBV ( hepatitis B infection) JAUNDICE, sort : centile EQvas _index DW1 DW2, centile(50)

--------------------------------------------------------------------------------------------------------------------

-> HBV = 0, JAUNDICE = 0

-- Binom. Interp. --

Variable | Obs Percentile Centile [95% Conf. Interval]

-------------+-------------------------------------------------------------

EQvas | 83 50 60 50 70

_index | 83 50 .695 .6400415 .695

DW1 | 83 50 .4 .3 .5

DW2 | 83 50 .305 .305 .3599585

--------------------------------------------------------------------------------------------------------------------

-> HBV = 0, JAUNDICE = 1

-- Binom. Interp. --

Variable | Obs Percentile Centile [95% Conf. Interval]

-------------+-------------------------------------------------------------

EQvas | 11 50 60 47.12727 70

_index | 11 50 .596 .35236 .6542982

DW1 | 11 50 .4 .3 .5287273

DW2 | 11 50 .404 .3457018 .64764

--------------------------------------------------------------------------------------------------------------------

-> HBV = 1, JAUNDICE = 0

-- Binom. Interp. --

Variable | Obs Percentile Centile [95% Conf. Interval]

-------------+-------------------------------------------------------------

EQvas | 7 50 80 70 86.85714

_index | 7 50 .695 .652 .787

DW1 | 7 50 .2 .1314286 .3

DW2 | 7 50 .305 .213 .348

Note that the group HBV = 1, JAUNDICE = 1 is not represented ( excluded)

Supplementary Table 3 Heteroskedastic linear regression output from Stata with EQ-VAS derived disability weights as the dependent variable.

| Heteroskedastic linear regression Number of obs = 101 | | |  |  |  |  |
| --- | --- | --- | --- | --- | --- | --- |
| **Two-step GLS estimation** |  |  |  |  |  |  |
| Wald chi2(6) = 141.48 | |  |  |  |  |  |
| Prob > chi2 = 0.0000 | |  |  |  |  |  |
|  |  |  |  |  |  |  |
|  |  |  |  |  |  |  |
| DW1 | Coef. | Std. Err. | z | P>z | [95% Conf. | Interval] |
|  |  |  |  |  |  |  |
| DW1 |  |  |  |  |  |  |
| acuteUGIB | 0.3014037 | 0.0350787 | 8.59 | 0 | 0.2326507 | 0.3701568 |
| pzq | 0.1477402 | 0.0384645 | 3.84 | 0 | 0.0723511 | 0.2231292 |
| ager1 | 0.0458407 | 0.0125901 | 3.64 | 0 | 0.0211646 | 0.0705168 |
| BMI185 | 0.0451096 | 0.0459034 | 0.98 | 0.326 | -0.0448595 | 0.1350786 |
| female | 0.0648672 | 0.0330839 | 1.96 | 0.05 | 0.0000239 | 0.1297105 |
| JAUNDICE | -0.1202298 | 0.0418105 | -2.88 | 0.004 | -0.2021769 | -0.0382828 |
| _cons | -0.1389374 | 0.0855517 | -1.62 | 0.104 | -0.3066157 | 0.0287409 |
|  |  |  |  |  |  |  |
| lnsigma2 |  |  |  |  |  |  |
| acuteUGIB | -0.5922417 | 0.5794634 | -1.02 | 0.307 | -1.727969 | 0.5434857 |
| pzq | 0.8166327 | 0.7337752 | 1.11 | 0.266 | -0.6215402 | 2.254806 |
| ager1 | -0.0204513 | 0.1942937 | -0.11 | 0.916 | -0.4012599 | 0.3603573 |
| BMI185 | 0.4982001 | 0.6126965 | 0.81 | 0.416 | -0.702663 | 1.699063 |
| female | -0.2705673 | 0.471245 | -0.57 | 0.566 | -1.194191 | 0.653056 |
| JAUNDICE | -0.2658229 | 0.7664471 | -0.35 | 0.729 | -1.768032 | 1.236386 |
| _cons | -5.001546 | 1.558837 | -3.21 | 0.001 | -8.05681 | -1.946282 |
|  |  |  |  |  |  |  |
| **Wald test of lnsigma2=0: chi2(6) = 3.73 Prob > chi2 = 0.7132** | | |  |  |  |  |

Supplementary Table 4 Linear regression output from Stata for Model 2 with EQ-5D disability weights as the dependent variable.

|  |  |  |  |  |  |  |
| --- | --- | --- | --- | --- | --- | --- |
| Linear regression Number of obs = 101 |  |  |  |  |  |  |
| F(8, 92) = 8.68 |  |  |  |  |  |  |
| Prob > F = 0.0000 |  |  |  |  |  |  |
| **R-squared = 0.4507** |  |  |  |  |  |  |
| **Root MSE = .16511** |  |  |  |  |  |  |
|  |  | Robust HC2 |  |  |  |  |
| DW2 | Coef. | Std. Err. | t | P>t | [95% Conf. | Interval] |
|  |  |  |  |  |  |  |
| BMI185 ( underweight) | 0.1866911 | 0.0695674 | 2.68 | 0.009 | 0.0485243 | 0.3248579 |
| acuteUGIB | 0.0996866 | 0.0412727 | 2.42 | 0.018 | 0.0177155 | 0.1816577 |
| ASCITEUS ( ascites) | 0.1854059 | 0.0700371 | 2.65 | 0.01 | 0.0463062 | 0.3245055 |
| ager1 ( age range by decade) | 0.0326022 | 0.0149231 | 2.18 | 0.031 | 0.0029637 | 0.0622407 |
| female | 0.1021255 | 0.0366122 | 2.79 | 0.006 | 0.0294105 | 0.1748405 |
| Pzq ( praziquantel) | 0.1202994 | 0.039513 | 3.04 | 0.003 | 0.0418232 | 0.1987757 |
| farmer | 0.0675535 | 0.0366485 | 1.84 | 0.069 | -0.0052335 | 0.1403405 |
| WHOZ ( anemia) | -0.0069236 | 0.017285 | -0.4 | 0.69 | -0.0412531 | 0.0274058 |
| _cons | -0.1181696 | 0.115862 | -1.02 | 0.31 | -0.3482815 | 0.1119424 |
|  |  |  |  |  |  |  |

Supplementary Table 5 linear regression output from Stata of depression, ascites, and hepatitis B status with EQ-VAS derived disability weights as the dependent variable.

| Linear regression Number of obs = 101 | |  |  |  |  |  |
| --- | --- | --- | --- | --- | --- | --- |
| F(3, 97) = 32.07 |  |  |  |  |  |  |
| Prob > F = 0.0000 | |  |  |  |  |  |
| R-squared = 0.2896 | |  |  |  |  |  |
| Root MSE = 0.18485 | |  |  |  |  |  |
|  |  | Robust |  |  |  |  |
| EQ-VAS derived disability weights | Coef. | Std. Err. | t | P>t | [95% Conf. | Interval] |
|  |  |  |  |  |  |  |
| Anxiety/depression | 0.1623694 | 0.0408926 | 3.97 | 0.001 | 0.0812088 | 0.24353 |
| Ascites | 0.1457435 | 0.0411942 | 3.54 | 0.001 | 0.0639843 | 0.2275027 |
| Hepatitis B surface antigen positive | **-0.1462674** | 0.0353656 | -4.14 | 0.001 | -0.2164583 | -0.0760765 |
| _cons | 0.0501 | 0.0797595 | 0.63 | 0.531 | -0.1082005 | 0.2084005 |
